# Androgen receptor status predicts development of brain metastases in ovarian cancers

**DOI:** 10.18632/oncotarget.17068

**Published:** 2017-04-12

**Authors:** Gloria Mittica, Rebecca Senetta, Giulia Scotto, Massimo Aglietta, Furio Maggiorotto, Eleonora Ghisoni, Sofia Genta, Renzo Boldorini, Claudia Manini, Isabella Morra, Roberta Buosi, Anna Sapino, Paola Cassoni, Giorgio Valabrega

**Affiliations:** ^1^ Department of Oncology, University of Turin, Turin, Italy; ^2^ Division of Medical Oncology-1, Candiolo Cancer Institute-FPO- IRCCS, Candiolo, Italy; ^3^ Unit of Pathology Candiolo Cancer Institute-FPO- IRCCS, Candiolo, Italy; ^4^ Division of Gynecologic Oncology, Candiolo Cancer Institute-FPO- IRCCS, Candiolo, Italy; ^5^ Department of Health Science, University of Eastern Piedmont “Amedeo Avogadro”, Novara, Italy; ^6^ Unit of Pathology, Giovanni Bosco Hospital, Turin, Italy; ^7^ Unit of Pathology, Città della Salute e della Scienza, Turin, Italy; ^8^ Division of Oncology, Santo Spirito Hospital, Casale Monferrato, Italy; ^9^ Department of Medical Sciences, University of Turin, Turin, Italy

**Keywords:** androgen receptor, ovarian cancer, brain metastases

## Abstract

Brain metastases are uncommon localizations in epithelial ovarian cancer (EOC), their reported incidence is increasing and no predictive biomarkers have been identified yet. Goals of this study were: i) to define a possible association between Estrogen Receptor (ER), Progesterone Receptor (PR), Androgen Receptor (AR), human EGF receptor 2 (HER2) and brain progression in EOC patients, and ii) to identify differences in ER, PR, AR and HER2 protein expression from primary EOC and its matched resected brain metastasis. A retrospective series of 11 EOC with matched brain metastasis surgically removed was collected. For comparison, a “Control dataset” of 22 patients, without evidence of brain involvement after an adequate follow up was matched. ER, PR, AR and HER2 *status* were analyzed by means of immunohistochemistry forCases (both primary and metastatic lesions) and Controls.

Univariate analysis showed that AR *status* was significantly associated with brain localization, both considered as discrete variable (cut-off: 10%, *p*=0.013) and as continuous one (*p*=0.035). Multivariate analysis confirmed this trend (*p*=0.053). When considered as continuous variables, ER and AR showed greater expression in primary tumors in comparison with brain metastases (*p*=0.013 and *p*=0.032, respectively).

In our series, AR predicts brain involvement, with a 9.5 times higher propensity for AR-negative EOC. Moreover, brain dissemination is probably the result of progressive dedifferentiation of primary tumor, shown by reduction of ER and AR expression in metastases. Further studies are required, in order to anticipate and improve multimodal treatment of brain metastases.

## INTRODUCTION

Epithelial ovarian cancer (EOC) is the first cause of death among women with gynaecological malignancies [1]. In its natural history, this tumor tends to remain localized in the abdomen and pelvis even in advanced stage of disease, whereas haematogenous spread is a late event [2]. In fact, distant metastases account for approximately 16% of cases, and pleura (33%), liver (26%) and lung (3%) are the most common sites of tumor progression [3]. Central nervous system (CNS) involvement is a very uncommon and late event with an incidence ranging from 0.29 to 12% according to different series [3–6]. More recently, an increase in metastatic CNS involvement has been reported [5, 7, 8],probably reflecting prolonged patients’ survival related to improved surgery, radiotherapy and medical treatments [3, 4, 6, 8–14]. Several favorable prognostic factors have been identified such as younger age at time of diagnosis, Karnofsky PS, absence of extracranial lesions or solitary brain lesion [4, 6, 8–14]. However, prognosis remains globally unfavourable with a median overall survival (OS) of about 9.6 months for patients treated with only best supportive care, and 20.5 months for those undergoing multimodal treatments [13]. Clearly, early detection of CNS involvement may enhance the possibility of successfully treating these patients.

Hormonal receptor *status* (Estrogen Receptor-ER, Progesterone Receptor-PR and Androgen Receptor-AR) and Epidermal growth factor receptor 2 (HER2) *status* have been widely investigated as potential prognostic parameters in EOC patients, but reported results are controversial [15–22].

The aims of our study were the following: i) to define a possible association between ER, PR, AR, andHER2 with CNS progression in EOC patients, and ii) to identify differences in ER, PR, AR and HER2 protein expression from primary EOCs and their matched resected brain metastases.

## RESULTS

### Patients

Case dataset. The clinical and pathological features of primary ovarian lesions of the Case dataset are summarized in Table 1. Briefly, patient's median age was 61 years and according to the FIGO classification there were 2/11 (18%) stage II, 6/11 (55%) stage III, and 3/11 (27%) stage IV patients. Up-front surgery was performed in 9 cases (82%) and in 7 patients (64%) a macroscopic residual tumor was assessed; most of the cases were high-grade serous adenocarcinoma with solid and papillary features (82%). All patients underwent adjuvant platinum-based chemotherapy. At the time of statistical analysis (June 2016), 3 of 11 (27%) patients were alive, whereas the remaining 7 patients (64%) had died; one patient was lost. The first site of relapse was brain in 7/11 (64%) cases, followed by lymph node (3/11, 27%); other sites included liver and mediastinum. All patients developed a CNS metastasis (median age: 62 years) with a median bPFS of 23 months (range 11-68). The majority of cases showed a single CNS metastasis (73%, 8/11) localized in cerebral hemispheres and, specifically, frontal lobe represented the most common localization (45%, 5/11). Surgical CNS treatment consisted of gross total or incomplete resection followed by systemic chemotherapy (Pegylated liposomal doxorubicin and Vinorelbine, oral Topotecan, Pegylated liposomal doxorubicin alone, Carboplatinum and Gemcitabine)in 4 patients (36%), loco-regional treatments as Whole Brain Radiotherapy (WBRT) in 64% (7/11) and stereotactic radiotherapy in 9% of cases (1/11). The bOS was 7 months (range 3-64). Table 2 reports the clinical parameters of the 11 cases with CNS metastases.

**Table 1:** Clinico-pathological features of primary ovarian lesion: “Case dataset” *vs*. “Control dataset”

Clinico-histopathological parameters	Case datasetN=11 (%)	Control datasetN=22 (%)	*p*
**Age, median (years) [range]**	61 [44–72]	65.5 [36–76]	0.323
**Histological type**			
Serous	9 (82)	22 (100)	0,12
Endometrioid	2 (18)	0 (0)	
**Histological grade**			
G2	1 (9)	2 (9)	1
G3	10 (91)	20 (91)	
**FIGO Stage**			
II	2 (18)	4 (18)	1
III	6 (55)	12 (55)	
IV	3 (27)	6 (27)	
**Type of surgery**			
Up-front	9 (82)	10 (45)	0.067
Neoadjuvant CT + IDS	2 (18)	12 (55)	
**Macroscopic residual tumor**			
Present	7 (64)	15 (68)	0.9
Absent	3 (27)	7 (32)	
Not available	1 (9)	0 (0)	
**First-line chemotherapy**			
Platinum-based	11 (100)	22 (100)	1
Other	0 (0)	0 (0)	
**Relapse**			
Present	11 (100)	17 (77)	0.143
Absent	0 (0)	5 (23)	
**First site of relapse**			
Brain	7 (64)	0 (0)	0.001
Lymph nodes and/or	3 (27)	15 (88)*	
peritoneum other	1 (9)	2 (12) *	
**Patient's *status***			
Alive	3 (27)	9 (41)	0.301
Dead	7 (64)	13 (59)	
Not available	1 (9)	0 (0)	
**PFS**, median (months) [range]	22 [7–50]	16 [4–73]	
**OS**, median (months) [range]	47 [17–110]	38.5 [6–82]	

* In 5 patients a progression disease was not evident.

IDS: Interval Debulking Surgery; CT: Chemotherapy; PFS: Progression Free Survival; OS: Overall Survival.

**Table 2:** Clinical parameters of 11 brain metastases included in the study

Cases	Age	bPFS (months)	Neurological symtoms	Number of brain lesion	Site of brain lesion	Treatment of brain metastases	bOS (months)
**1**	49	11	NA	1	Parietal lobe	Surgery, Chemotherapy	6
**2**	57	68	Confusional state	1	Occipital lobe	Surgery	42
**3**	70	29	Ataxia	1	Parieto-occipital lobe	Surgery WBRT	41
**4**	70	22	Unilateral symptoms	1	Parietal lobe	Surgery Chemotherapy WBRT	6
**5**	50	18	Headache, vertigo	2	Temporo-occipital lobe, frontal lobe	Surgery Chemotherapy WBRT	29
**6**	70	28	Aphasia, disorientation, dizziness	1	Parietal lobe	Surgery, stereotactic radiotherapy	56
**7**	52	54	NA	1	Frontal lobe	Surgery Chemotherapy WBRT	7
**8**	62	15	Headache, altered walking gait	5	Temporal lobe, frontal lobe, occipital lobe	Surgery WBRT	3
**9**	46	23	NA	1	NA	Surgery	3
**10**	72	18	Ataxia, dysmetria	6	Frontal lobe (the major)	Surgery WBRT	7
**11**	74	25	Vertigo	1	Frontal lobe	Surgery WBRT	64

NA: not available; WBRT: Whole Brain Radiotherapy; bPFS: Progression Brain Metastasis Free Survival; bOS: Brain Metastasis Overall Survival.

Control dataset. This subgroup included 22 patients with a median age at primary ovarian cancer diagnosis of 65.5 years (range 36-76). At diagnosis, 12/22 presented a FIGO stage III (55%), 6/22 (27%) a FIGO stage IV and 4/22 (18%) a FIGO stage II. All patients had a histological diagnosis of serous ovarian cancer. Twelve out of 22 (55%) cases underwent neoadjuvant platinum-based chemotherapy (NACT) followed by Interval Debulking Surgery (IDS); the remaining cases had up-front surgery; in 15/22 patients (68%) the surgery resulted suboptimal with a macroscopic residual tumor. All patients underwent adjuvant platinum-based chemotherapy. Seventeen (77%) patients experienced a progression disease with a median PFS of 16 months (range 4-73). Specifically, the most frequent sites of relapse were peritoneum and/or lymph nodes (88%, 15/17). The OS was 38.5 months (range 6-82) and, at the time of statistical analysis, 13/22 (59%) patients were dead. Table 1 resumes the main clinico-pathological features of Control dataset.

The two subgroups (*Case* and *Controls*)resulted homogeneous and comparable. There were not statistically significant differences for age (p = 0,323), tumor grade (*p*=1), stage of disease (*p*=1), type of surgery (p= 0,067), histotype (*p*=0,12) residual disease after surgery (*p*=0.9), (Table 1).

### Immunohistochemical results

All cases of both datasets were evaluated by immunohistochemistry, but we had necessarily to exclude one case of primary ovarian cancer in the Case dataset as patient underwent NACT with a good pathological tumor response (only a sub-millimetric focus of residual carcinoma was found on histological specimen, insufficient to correctly evaluate immune-histochemical analyses). Table 3a reports immune-histochemical results of *Case* (both primary and CNS metastasis) and Control dataset, considered as dichotomized variables, whereas Table 3b and Table 4 as continuous variables.

**Table 3A:** Immunohistochemical results for hormonal receptors (ER, PR, AR categorised as dichotomised variables) and HER2 in Case dataset (both primary and metastatic lesions) and Control dataset: statistical analysis

Immunohistochemistry	Cut-off	Case dataset-primary ovarian tumorsN/10* (%)	Case dataset-brain metastasesN/11 (%)	Control dataset- primary ovarian tumorsN/22 (%)	*p* (Cases *vs*. Controls:IHCcomparison)	OR (Cases *vs*. Controls)(IC 95%)	*p* (Cases: primary *vs*. metastatic lesions: IHC comparison)
***ER***	**< 1%**	1 (10)	2 (18)	1 (5)	*p*=0.534		*p*=0.200
	**≥ 1%**	9 (90)	9 (82)	21 (95)			
	**< 10%**	1 (10)	3 (27)	2 (9)	*p*=1.000		*p*=0.300
	**≥ 10%**	9 (90)	8 (73)	20 (91)			
	**IRS ≤ 2**	1 (10)	4 (36)	2 (9)	*p*=1.000		*p*=0.400
	**IRS > 2**	9 (90)	7 (64)	20 (91)			
***PR***	**< 1%**	7 (70)	9 (82)	11 (50)	*p*=0.446		*p*=1.000
	**≥ 1%**	3 (30)	2 (18)	11 (50)			
	**< 10%**	7 (70)	10 (91)	13 (59)	*p*=0.703		*p*=0.300
	**≥ 10%**	3 (30)	1 (9)	9 (41)			
	**IRS ≤ 2**	7 (70)	10 (91)	13 (59)	*p*=0.703		*p*=0.300
	**IRS > 2**	3 (30)	1 (9)	9 (41)			
***AR***	**< 1%**	3 (30)	6 (55)	2 (9)	*p*=0.293		*p*=1.000
	**≥ 1%**	7 (70)	5 (45)	20 (91)			
	**< 10%**	6 (60)	8 (73)	3 (14)	***p*=0.013**	9.5 (1.64 - 54.99	*p*=0.133
	**≥ 10%**	4 (40)	3 (27)	19 (86)			
	**IRS ≤ 2**	7 (70)	10 (91)	10 (45)	*p*=0.265		*p*=0.300
	**IRS > 2**	3 (30)	1 (9)	12 (55)			
***HER2***	**0**	9 (90)	7 (64)	14 (63)	*p*=0.069	1.6 (0.15 - 17.22)	*p*=0.364
	**1+**	0 (0)	3 (27)	5 (23)			
	**2+**	0 (0)	1 (9)	3 (14)			
	**3+**	1 (10)	0 (0)	0 (0)			

* one case was excluded due the limited neoplastic tissue available.

ER: Estrogen Receptor; PR: Progesterone Receptor; AR: Androgen Receptor; HER2: Human Epidermal Growth Factor Receptor 2; IRS: Immunoreactive Score; OR: Odds Ratio.

**Table 3B:** Immunohistochemical results for hormonal receptors (ER, PR, AR considered as continuous variables) in Case dataset and Control dataset: statistical analysis

Immunohistochemicalparameters	Case *vs*. Controldataset	N	Mean	Median	Range	*p*
**ER**	*Cases*	10	57.4	62.5	0-95	*p*=0.397
	*Controls*	22	67.1	75	0-95	
**PR**	*Cases*	10	6.5	0	0-35	*p*=0.442
	*Controls*	22	11.5	0.5	0-75	
**AR**	*Cases*	10	16.9	6.5	0-65	***p*=0.035**
	*Controls*	22	40.5	32.5	0-90	

ER: Estrogen Receptor; PR: Progesterone Receptor; AR: Androgen Receptor.

**Table 4:** Immunohistochemical results for hormonal receptors (ER, PR, AR considered as continuous variables) in the Case dataset - primary *vs*. metastatic lesions: statistical analysis

Immunohistochemicalparameters	Primary tumor *vs*. brainmetastasis	Mean	Median	Range	*p*
**ER**	Primary tumor	57.4	62.5	0-95	***p*=0.013**
	Brain metastasis	34	22.5	0-76	
**PR**	Primary tumor	6.5	0	0-35	*p*=0.396
	Brain metastasis	3.1	0	0-30	
**AR**	Primary tumor	16.9	6.5	0-65	***p*=0.032**
	Brain metastasis	6.8	0	0-35	

ER: Estrogen Receptor; PR: Progesterone Receptor; AR: Androgen Receptor.

**Figure 1:**
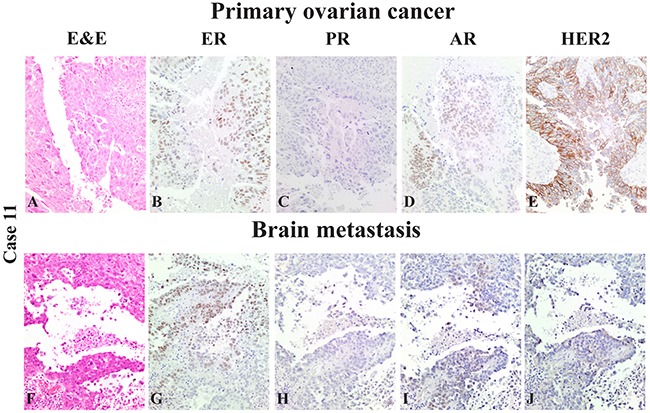
Case 11 of the “Case dataset”: representative case of a serous high-grade ovarian cancer (A-E) (A: H&E, 10x): hormonal receptor *status* (B: ER=60%, 10x; C: PR=0%, 10x; D: AR=8%, 10x) and HER2tumor expression (E: HER2 3+, 10x) Its derived brain resected metastasis **(F-J)** with the same morphological appearance (F: H&E, 10x): hormonal receptor *status* (G: ER=72%, 10x; H: PR=0%, 10x; I: AR=5%, 10x) and HER2 metastasis expression (J: HER2 2+, 10x).

Figure 1 shows the immune-histochemical profile of a representative case of serous ovarian carcinoma included in the Case dataset (*Case 11, Table 2*) and its matched brain metastasis of one.

### Statistical analysis

Case dataset vs. Control dataset. Using a cut-off of 10%, only AR showed a significant difference of expression (*p*=0.013) between the two studied series: the risk to develop a brain metastasis appeared 9.5 times greater in patients with AR-negative primary ovarian cancer (Table 3a). Although at the limit for a statistical significant correlation, the HER2 expression in primary ovarian lesions was lower in patients who developed brain metastasis (*p*=0.069) (Table 3a). When considered as a continuous variable, AR retained its predictive role (mean *Case dataset*: 16.9; mean Control dataset: 40.5; *p*=0.035), as mainly expressed in primary ovarian tumors in the Control dataset (Table 3b). None of the other immune-histochemical biomarkers proved to be statistically significant (Table 3a and 3b). Cox multivariate analysis confirmed the lack of AR tumor expression in primary ovarian lesions as a negative independent prognostic parameter, supporting brain metastasis progression (*p*=0.053, CI 95% 1.000-1.073).

**Figure 2:**
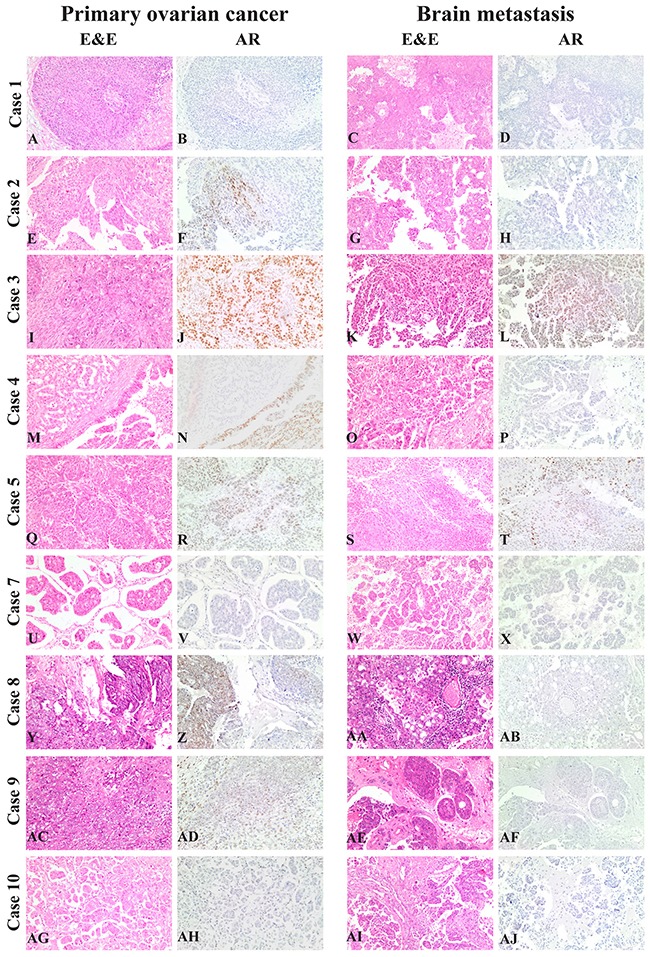
AR tumor expression in primary and metastatic lesions of our “Case dataset” From Case 1 to Case 10 (**A, C**: H&E, 10x/B-D: AR, 10x, Case 1; **E, G**: H&E, 10x/F, **H**: AR, 10x, Case 2; **I, K**: **H&E**, 10x/J, L: AR, 10x, Case 3; **M, O**: H&E, 10x/N, **P**: AR, 10x, Case 4; **Q, S**: H&E, 10x/R, **T**: AR, 10x, Case 5; **U, W**: H&E, 10x/V, **X**: AR, 10x, Case 7; **Y**, **AA**: H&E, 10x/Z-, **AB**: AR, 10x, Case 8; **AC**, **AE**: H&E, 10x/AD, **AF**: **AR**, 10x, Case 9; **AG, AI**: H&E, 10x/AH, **AJ**: AR, 10x, Case 10). Case 6 is not showed as excluded from the immunohistochemical analysis, due to insufficient residual cancer after neoadjuvant chemotherapy.

*Primary ovarian cancers vs. matched brain metastases*. No statistical differences were observed when biomarkers were considered as dichotomized variables (Table 3a). However, if considered as continuous variables, ER and AR showed a statistically significant greater expression in primary tumors in comparison with brain metastases (*p*=0.013, CI 95%=6.16-40.65 and *p*=0.032, CI 95%=1.12-19.08 respectively) (Table 4). Figure 2 shows the immunohistochemical profile of AR in primary and metastatic brain lesions of our “Case dataset”.

## DISCUSSION

Currently small retrospective studies suggest that histology, stage of disease [13], loss of BRCA1 function [23], MDR-1 tumor expression [24] may be associated with CNS progression in very small series of patients. In the present study we investigated the predictive role of AR, ER, PR and HER2.

Hormone receptor *status* has been extensively investigated in EOC patients, playing an important role in ovarian cancer pathogenesis [25], but the literature data appear still conflicting and above all not conclusive [15–22]. In a recent review, including 35 studies investigating the prognostic role of hormone receptors in EOC, ER failed to predict patients’ outcome, whereas elevated levels of PR and HER2 predicted favorable and worse survival, respectively [26]. AR tumor expression has been recently suggested to be a favourable prognostic parameter in serous EOCs, especially when co-expressed with PR [27, 28]. Notably, AR is the only hormone receptor included in the five good-prognosis predictors of the protein-driven index of ovarian cancer (PROVAR), a protein-based panel able to predict time to EOC recurrence [29]. Due to the limited number of events, in the subgroup of EOC patients who developed CNS progression, hormonal receptor *status* role has not yet widely been explored.

In our case-control study, comparing patients who developed a CNS metastasis and patients who did not, we highlight a predictive role of AR. Specifically, patients with AR-negative EOC show a 9.5 times greater propensity to develop CNS metastases than AR-positive EOC (cut-off: 10%); moreover, when considered as continuous variable, AR retains its predictive value. Multivariate analysis confirms reduced AR expression in primary EOC as a negative independent predictive parameter. In a previous study, AR *status* has been reported to be associated with a prolonged ovarian cancer specific survival, underlining its favourable prognostic role [27], but our paper, for the first time, describe its predictive value in the subgroup of EOC metastatic to the brain. In the literature, different cut-off values have been considered in assessing the hormone receptor *status* positivity [16, 17, 22, 27, 28, 30], reason that could partially explain the discrepancy observed among the different works. Thus, to bypass this impasse and achieve a clearer view of the data, we consider different cut-off of positivity, according to literature and in particular 1%, 10% or IRS [16–22, 27, 28, 30]. The AR cut-off point of 10% in primary ovarian lesion seems to be the best predictor of brain progression in EOC patients. Therefore, EOC primary tumors that express AR are lower prone to cerebral spread. This observation may be in agreement with other recent studies stressing the favourable prognostic role of AR identifying a subgroup of patients with better survival in ovarian, breast and endometrial cancers, founding the basis to an endocrine anti-AR therapeutic approach [27, 28, 31–33].

In our study, a low or none tumor expression of HER2 identifies a subgroup of EOC patients with higher risk to develop brain metastases even if with a borderline statistical significance, probably related to the small number of analysed cases. In general, very few data are reported in literature regarding HER2 prognostic/predictive role in EOC, although its overexpression seems to be related to worse patient's outcome [30, 34, 35]. In this setting, our observation could seem in contrast with previously reports [26, 30, 34, 35], but we might speculate that as brain progression represent a rare and late event in EOC natural history [13], it could be possible that patients with HER2 overexpression die before brain metastatic dissemination.

Almost no data are reported in literature about protein expression profile assessment in brain metastases from EOC: the only, to our knowledge, is the paper by Yoshida *et al*. including a single case of matched primary and metastatic lesion [36]. The reason for this scarcity may rely on both the rarity of the event and of the surgery, as only 30% of patients [3, 13] undergo brain metastatic resection. Thus, our paper is the first reported work that focused on investigation of protein expression profile in a consecutive case series of EOC and their matched brain metastases. Considering hormone receptors *status* as continuous variables, we observed a significant difference between primary and brain lesions in expressing ER(57.4% *vs*. 34%, *p*=0.013) and AR (16.9% *vs*. 6.8%, *p*=0.032). Specifically, a reduction of both receptors has been detected in metastatic tissue. Since CNS progression is clearly an adverse prognostic feature, our results are consistent with previously reported good prognostic role of AR, as the tumors seem to lose AR protein expression during the metastatic spread to brain. A progressive ‘de-differentiation’ of neoplastic cells could be hypothesized.

In conclusion, the increased incidence of CNS metastases from EOC underlines the importance of identifying predictive biomarkers tightly associated with CNS progression. The identification of patients at higher risk to develop CNS metastases may help to improve prognosis and quality of life, as previously extensively reported [3, 4, 6, 8, 10, 11, 13]. Although the present case-control study is the biggest ever reported in literature, in terms the number of enrolled patients and the possibility to analyse EOC and their matched brain metastasis, a validation in larger series is likely.

## MATERIALS AND METHODS

### Patient's collection

From a consecutive, retrospective and multi-institutional series of 1092 patients who underwent surgery for brain metastases resection between January 1998 and December 2013, a cohort of 24 patients with CNS metastases developed from a primary, histologically confirmed EOC was extrapolated. The initial cohort was retrieved from the Pathology archives of the Hospitals included in the Neuro-Oncological Network of the Piedmont Region-Italy (AOU Città della Salute e della Scienza of Turin, AO San Giovanni Bosco Hospital of Turin, AOU Maggiore della Carità of Novara, AO S.Croce e Carle Hospital of Cuneo, AO SS. Antonio, Biagio and Cesare Arrigo of Alessandria). Essential inclusion criteria for the study were the availability of both i) paraffin-embedded tissue blocks (corresponding to primary ovarian lesion and its matched brain metastasis) and ii) follow-up data, thus 13/24 cases were excluded; therefore, a series of 11 patients was finally collected representing our “Case dataset”. For each patient, clinico-histopathological data were obtained by medical records. The following parameters were recorded: i) age at diagnosis; ii) date of primary tumor and CNS metastasis diagnosis; iii) morphological features of ovarian and brain metastasis as tumor histotype and grade (according to World Health Organization Classification of Tumours of Female Reproductive Organs, 4th Edition); iv) date and site of first relapse, v) type of patient's treatment; vi) date of death or last follow up (FU). OS was determined as the time from the date of EOC diagnosis to the date of patient death or last FU, whereas progression free survival (PFS) as the time from the EOC diagnosis to the date of first clinical relapse. In addition, we calculated the Progression Brain Metastasis Free Survival (bPFS) as the time from the date of ovarian cancer first diagnosis to brain metastasis, and the Brain Metastasis Overall Survival (bOS) as the time from the date of brain metastasis diagnosis to death or last FU.

For comparison, we evaluated a second series of 22 cases, named “Control dataset”, extracted from the clinical records of Candiolo Cancer Institute, including patients with a diagnosis of ovarian cancer who had not developed brain metastasis after a median FU of 38,5 months (range 6-82 months). In order to statistically compare the two subgroups and to avoid confounders, *Cases* were paired with *Controls* with a 1:2 *ratio*, according to the following three clinico-pathological parameters: tumor grade, clinical tumor stage and residual disease after surgery (present *vs*. absent). The same clinico-morphological features collected for the Case dataset were also obtained for this subgroup of patients.

The study was submitted to and approved by the Ethic Institutional Review Board for “Biobanking and use of human tissues for experimental studies” of the Pathology Service of the AOU Città della Salute e della Scienza (Turin, Italy). The project provided a verbal and not written informed consent from the patients due to the retrospective approach of the study, which did not impact on their treatment. All the cases were anonymously recorded. The Institutional Review Board approved this consent procedure.

### Immunohistochemistry procedures

The most representative paraffin block was selected for each lesion and immune-histochemistry was performed in all cases. Three-micrometer-thick serial paraffin sections were prepared and routinely stained with Hematoxylin and Eosin (H&E); additional sections, collected on superfrost plus slides, were used for immune-histochemical analysis. Immune-histochemical reactions using antibodies anti-ER (monoclonal antibody, clone SP1, pre-diluted, Ventana, Roche); anti-PR (monoclonal antibody, clone 16, diluted 1:100, Novocastra™ Leica); anti-AR (monoclonal antibody, clone SP107, pre-diluted, Ventana, Roche), and anti-HER2 (rabbit monoclonal antibody, clone 4B5, pre-diluted, Ventana, Roche) were performed in an automated immunostainer (VentanaBenchMark XT AutoStainer, Ventana Medical Systems, Tucson, AZ, USA). Appropriate positive and negative controls were included for each immune-histochemical run.

### Staining interpretation

Two observers, who were blinded to clinical data, independently evaluated the staining results. Hormone receptors were considered as follows: i) continue variable (number of positive neoplastic cells considered as a percentage ranging from 0 to 100%), ii) discrete variable using cut-off of 1% [30, 31, 37] and 10% [16, 17, 22, 27, 28, 38] as previously reported in literature, and iii) dichotomised variable according to the recently reported Immunoreactive Score (IRS) [18–21]. HER2 *status* was assessed according to ASCO/CAP recommendations (2013) for breast cancer [39].

### Statistical analysis

All statistical analyses were performed using SPSS software for Windows (version 22.0; SPSS Inc., Chicago, IL). Quantitative variables were initially compared with Pearson Chi-square test, but due to the small number of cases results are not reliable. Therefore Fisher exact test has been considered for the further statistical analyses. Qualitative variables were compared using analysis of variance (ANOVA) or dependent T test for paired samples. Multivariate analysis was performed using regression analysis. P values <0.05 were considered significant, and all tests were two-tailed.
